# Making waves: Xanthates on the radar – Environmental risks and water quality impact

**DOI:** 10.1016/j.wroa.2024.100232

**Published:** 2024-07-03

**Authors:** Daniel J. Duarte, Renske P.J. Hoondert, Elvio D. Amato, Milou M.L. Dingemans, Stefan A.E. Kools

**Affiliations:** a KWR Water Research Institute, Nieuwegein, the Netherlands; b Institute for Risk Assessment Sciences, Utrecht Universities, Utrecht, the Netherlands

**Keywords:** Xanthates, Water quality, Human health risk, Environmental risk

## Abstract

Xanthates, derivatives of xanthic acid, are widely utilized across industries such as agrochemicals, rubber processing, pharmaceuticals, metallurgical, paper and mining to help separate metals from ore. Despite their prevalent use, many registered xanthates lack comprehensive information on potential risks to human health and the environment. The mining sector, a significant consumer of xanthates, drives demand. However, emissions into the environment remain poorly understood, especially concerning water quality. A recent EU parliamentary voting on water legislation highlights the urgency to address water pollution and the potential toxicity of xanthates. While limited data exist on xanthate presence in the environment, existing studies indicate their toxicity and contribution to environmental pollution, primarily due to carbon disulfide, a decomposition product. Concerns are mounting over the release of xanthates and carbon disulfide, particularly in mining areas near populated regions and river tributaries, raising questions about downstream impacts and public health risks. Proposed expansions of xanthate-reliant mining activities in Europe, heighten concerns about emissions and water quality impacts. Current databases lack xanthate-related monitoring data, hindering environmental and health risk assessments. Addressing this gap requires water sampling and chemical analysis and investigations into the use, occurrence, and potential impacts of xanthates from industrial activities on water bodies, including those used for drinking water production is imperative.

## Graphical abstract

**Figure:**
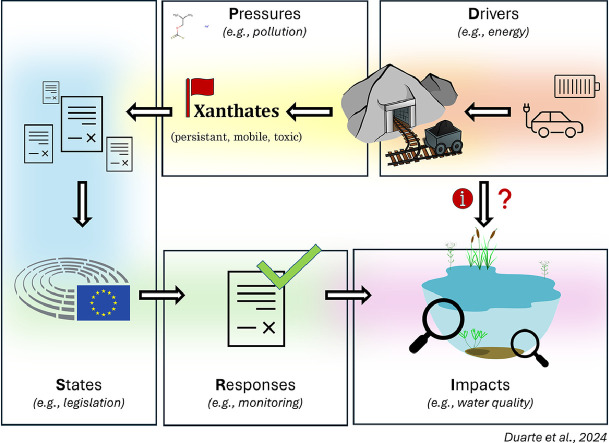

## Introduction

Recently, a voting session at the European Parliament took place (EP, 2023b), where several amendments to water legislation were adopted. Xanthates, organic reagents that are used for the flotation concentration and extraction of various minerals, are explicitly mentioned in the document, reading “*Xanthates should be included in the surface water watch list*”. Sulfates, another group of sulfur-containing compounds, were also proposed for inclusion. Furthermore, on November 28th, 2023, the Parliament and Council reached a provisional political agreement on the revision of the industrial emission directive (IED) and the directive on the landfill of waste and the new regulation on the Industrial Emissions Portal. The aim is to further reduce air, water, and soil pollution from large agro-industrial installations, to prevent health problems. If the agreement is adopted, environmental obligations will apply to extractive industry installations (mines), and strict achievable emissions levels will become mandatory (EP, 2023a). The novelty of xanthates lies in their context within EU regulations, their absence from environmental occurrence databases (e.g., NORMAN's EMPODAT database), potential toxicity to humans and water pollution, limited scientific literature despite regulatory developments, wide applications in industries such as agrochemicals, rubber processing, pharmaceuticals, metallurgical, paper and mining. The scarcity of public investigation, particularly from a water quality perspective, is noted, though a few publications suggest ongoing activities. These generally raise concern about expanding xanthate-reliant mining activities in European regions and questions whether there are water quality implications that require further investigation.

## Xanthates

Xanthic acids, or O-esters of dithiocarbonic acid (Moss et al., 1995), are compounds having the structure ROC(=S)SH. Xanthates are salts and esters of xanthic acid with general formula ROCS2-M^+^ where R and M indicate organic substituents and metallic ions (e.g., sodium or potassium), respectively (Qiao et al., 2023). Since the beginning of the 20th century, xanthates (O-alkyl dithiocarbonates) have been widely used as common organic reagents for the flotation concentration and extraction of a variety of hydrophobic sulfide and oxide minerals (Milosavljević et al., 2020; Yuan et al., 2023). More recently, xanthates have also shown potential for applications in colloidal science and nanotechnology, as capping/protecting agents for stabilizing silver, copper, gold and platinum colloids, and as precursors for metal sulfides (Efrima and Pradhan, 2003). Sufficient empirical hazard data are lacking in the scientific literature. As of 7th December 2023, there are 39 xanthates in ECHA's chemicals database. Of these, only 17 contain hazard information in the Substance InfoCard (ECHA, 2023), the vast majority of which pose a number of hazards, including to human health and the environment (Table 1).

**Table 1:** Xanthates with hazard classification. All data was sourced from ECHA (2023), except bioconcentration factor values sourced from EPA (2024).

Name	EC / List Number	Cas Number	Molecular formula	Molecular mass [g/mol]	Log Kow	Koc [L/kg]	Bioconcentration factor [L/Kg]	Hazard classification	Estimated tonnage band
((Ethoxythiomethyl)thio)acetic acid	623-864-6	25554-84-1	C5H8O3S2	180.24	0.787	200	3.47	Health hazard	?
3-methylbutoxymethanedithioic acid	807-374-1	2540-36-5	C6H12OS2	164.28	2.43	43.7	14.8	Serious health hazard Flammable Corrosive Hazardous to the environment	
Carbonic acid, dithio-, O-propyl ester, potassium salt	679-760-6	2720-67-4	C4H7KOS2	174.32	0.368	60.3	12.9	Health hazard	1-10
O,S-diethyl dithiocarbonate	210-813-4	623-79-0	C5H10OS2	150.25	2.01	64.6	10.5	Health hazard	?
O-butyl hydrogen dithiocarbonate, sodium salt	205-481-2	141-33-3	C5H9NaOS2	172.24	0.979	56.2	17.8	Health hazard	?
Potassium isopentyl dithiocarbonate (PIAX)	213-180-2	928-70-1	C6H11KOS2	202.37	1.90^.^	43.7^.^	14.8	Acute toxicity Flammable Corrosive Hazardous to the environment	1000-10000
Potassium O-(octahydro-4,7-methano-1H-inden-5-yl) dithiocarbonate	280-379-9	83373-60-8	C11H17KOS2	268.47	1.86	3470^.^	135	Health hazard	?
Potassium O-butyl dithiocarbonate	212-808-2	871-58-9	C5H9KOS2	188.34	0.909	56.2	17.8	Health hazard	?
Potassium O-ethyl dithiocarbonate (PEK)	205-439-3	140-89-6	C3H5KOS2	160.29	-0.226	20.9	1025 (exp.), 7.76 (est.)	Serious health hazard Health hazard Flammable Corrosive	1000-10000
Potassium O-hexyl dithiocarbonate (PHX)	220-331-6	2720-76-5	C7H13KOS2	216.4	1.77	91.2	85.1	Health hazard Flammable	Intermed. use
Potassium O-isobutyl dithiocarbonate (PIBX)	235-837-2	13001-46-2	C5H9KOS2	188.34	0.834	58.9	11	Serious health hazard Health hazard Flammable Corrosive	1000-10000
Potassium O-pentyl dithiocarbonate (PAX)	220-329-5	2720-73-2	C6H11KOS2	202.37	1.41	46.8	33.9	Health hazard Acute toxicity Flammable Corrosive Hazardous to the environment	1000-10000
Proxan-potassium (PIPX)	205-441-4	140-92-1	C4H7KOS2	174.32	0.288	49	11.2	Health hazard	?
Proxan-sodium (SIPX)	205-443-5	140-93-2	C4H7NaOS2	158.21	0.423	49	11.2	Serious health hazard Health hazard Flammable Corrosive Hazardous to the environment	1000-10000
Sodium O-ethyl dithiocarbonate (SEX)	205-440-9	140-90-9	C3H5NaOS2	144.18	-0.021	20.9	7.76	Serious health hazard Health hazard Flammable Corrosive Hazardous to the environment	1000-10000
S-allyl O-pentyl dithiocarbonate	220-977-9	2956-12-9	C9H16OS2	204.35	3.86	427	123	Health hazard Acute toxicity Flammable	100-1000
Sodium O-isobutyl dithiocarbonate (SIBX)	246-805-2	25306-75-6	C5H9NaOS2	172.24	1.65	58.9	11	Serious health hazard Health hazard Flammable Corrosive Explosive Hazardous to the environment	1000-10000

The hazardous properties of xanthates (salts of O-alkyldithiocarbonic acids) led to their banned use in cosmetic products marketed for sale or use in the European Union (ECHA, 2023). Xanthates are however used in many other applications such as agrochemicals (e.g., pesticides and antifungal agents), rubber processing (e.g., rubber vulcanization), medicines (e.g., against oxidative stress), metallurgy (e.g., metal protection and plating) and mining. The xanthate global market is expected to reach 874.3 billion US dollars by 2031 with an expected Compound Annual Growth Rate (CAGR) rise of 6% (TransparencyMarketResearch, 2023). Xanthates are used in many applications, but the mining and ore processing industry is expected to be the main driving factor in the rise in demand for xanthates, namely for the extraction of nickel, copper, zinc, lead, silver, gold, and sulfide minerals.

## Environmental emission

During the flotation process, approximately 50% of xanthates are consumed in the process, while the remnants are discharged with the flotation wastewater (Qiao et al., 2023). The excess and unreacted portion, estimated at hundred thousand tons annually, is emitted into tailing dams or drained by flotation plant effluent to surrounding water bodies and land (Prudence et al., 2022). This poses a risk, as leakages from tailing and tailing dam failure constitute the most mining-reported major incidents, 90% of which take place in active mines (Prudence et al., 2022). In fact, environmental problems associated with flotation reagents in mineral processing water have been well documented for decades (Chockalingam et al., 2003). A variety of treatment technologies are available to degrade xanthate pollutants in wastewater as to achieve safer discharge standards, although many technological limitations remain (Yuan et al., 2023). Still, challenges remain in the mitigation of the environmental impacts of these increasingly used chemicals and other polluting remnants (such as sulfates and polychlorinated biphenyls).

### Environmental fate

Xanthates degrade to inorganic sulfides or organo-sulfur compounds. In neutral or mildly alkaline solutions, sodium O-ethyl dithiocarbonate (SEX) degrades to the organic alcohol, carbon disulfide, sodium carbonate and sodium trithiocarbonate (NICNAS 1995). The half-life of SEX at pH 7 (25 °C) is around 260 h. (approx. 10 days) and doubles at above pH 8. Lower temperatures also extend the degradation of xanthates (Suvela, 2023). Xanthates are expected to last a few days in the aquatic environment (NICNAS 1995), are not expected to accumulate in biota (AGI, 2000) and their aquatic mobility is likely high (log Koc < 3), exacerbated by ionized states. Xanthates can last relatively longer in water bodies (e.g., sodium isoamyl xanthate) that offer neutral or weakly alkaline conditions. In this situation, they undergo slow hydrolysis, a significant process in determining the environmental fate of xanthates. For example, xanthic acids are moderately strong acids (e.g., butyl xanthic acid, pKa=2.23) which under alkaline, neutral and weakly acid conditions lead to xanthic acid dissociates (Shen et al., 2016). Although xanthates degrade slowly, these are not expected to accumulate in biota due to their ionic nature. The decomposition of xanthates can lead to a range of potentially hazardous degradation products, such as carbon disulfide (CS_2_), carbonyl sulfide (COS), alcohol (R-OH), carbonate (CO_3_^2−^), and hydrogen sulfur (HS) (Prudence et al., 2022). Currently, there is no chemical occurrence information on the xanthates chemical group in the EMPODAT database (https://www.norman-network.com/nds/empodat/), one of the most comprehensive databases of geo-referenced monitoring and bio-monitoring data on emerging substances (NORMAN, 2024).

### Analytical methods

Comprehensive data on the occurrence and environmental fate of xanthates and derivatives in aquatic environments and drinking water sources are largely missing. As new monitoring efforts will likely follow from the adoption of the new Watchlist, there may soon exist a pressing need for best-practice, cost-effective and easy-to-adopt analytical methods to facilitate this (Belkouteb et al., 2023). Mass spectrometry methods such as HPLC-MS/MS and ICP-MS/MS can be optimized and used to analyze water samples for xanthates and derivatives. For example, Qiao et al. (2023) have recently succeeded in measuring xanthates below the µg/L range in environmental samples by coupling HPLC with electrospray ionization (ESI) MS. Yet, few selective methods have been developed for xanthate(-derived) species and efficient sample preparation remains a challenge for the determination of low xanthate concentrations due to severe oxygen interference in sulphur readings (Suvela, 2023). Simpler methods such as UV/VIS spectrophotometry and flow injection analysis (FIA) could also be considered to expedite the generation of monitoring data considering the distinct absorption spectrum of xanthates (direct measure) and CS_2_ (indirect measure), as previously implemented by Muzinda and Schreithofer (2018). A handful of other non-selective methods exist (Suvela, 2023), although headspace gas chromatography with an electron capture detector (HS-GC-ECD) used in surface and drinking water has displayed great potential with a detection limit of 0.3 µg/L for potassium O-butyl dithiocarbonate (Li et al., 2015). The xanthates of interest and sample conditions will determine the selection and reliability of analytical methods.

## Aquatic toxicity

Acute toxicity of xanthates to freshwater fish and invertebrates has been studied as early as 1972 (e.g., Alto et al. (1977). Xanthates have been known to impair embryo development of *Salmonidae* fish at 30 µg/L, and cause embryo malformation of loach fish species at 56 µg/L (due to butyl xanthate), growth inhibition of algae *Scenedesmus* and *Chlorella pyrenoidosc*, and high mortality of fish and *Lemna minor* (duckweed) at concentrations > 5 mg/L (Qiao et al., 2023). The environmental effects of xanthates can be exacerbated by their ability to improve the mobility and bioavailability of sulfophilic metals (e.g., lead, cadmium, nickel) via complexation (Li et al., 2021; Prudence et al., 2022; Qiao et al., 2023). For example, potassium O-pentyl dithiocarbonate (PAX) increases the accumulation of cadmium (Cd^2+^) in trout by 2–8 times (Qiao et al., 2023). According to the Australian Government, insufficient data were available to derive reliable xanthate default guideline values for aquatic ecosystems (AGI, 2000). This lack of data still seems to be largely the case today (Fischer, 2023). Assuming that currently available ecotoxicological data gathered by Fischer (2023) is relevant and reliable, the PNECs for PAX, SEX, proxan-sodium (SIPX) and sodium O-isobutyl dithiocarbonate (SIBX) can be coarsely estimated to be 15.8, 4.7, 4.34 and 0.33 µg/L, respectively. Measured environmental concentrations are scarce, yet Qiao et al. (2023) found xanthates in environmental water samples at concentrations between 16.9 and 0.13 µg/L. This would suggest a potential risk to freshwater ecosystems. However, the existing information is dubious, and many knowledge gaps remain, rendering a current comprehensive risk assessment of xanthates and transformation products in waters undoable. Alternatively, Australia established low reliability trigger values of 0.05 µg/L for PIPX, SEX and SSBX (Sodium O-sec-butyl dithiocarbonate, CAS no. 36551-21-0) to 500 µg/L for PHX.

## Human exposure and toxicity

Scarce information is readily available about the presence of xanthates in drinking water sources. However, a few publications indicate that these chemicals are potentially toxic to humans (Bach et al., 2016; Elizondo-Álvarez et al., 2021) and severe water pollution can occur (Wang et al., 2023) in certain circumstances. Most concerns expressed in the scarce literature relate to the degradation product, carbon disulphide. Carbon disulfide (CS_2_) is the main degradation product of xanthates. It is a compound naturally present in the environment but with increasing occurrence due to anthropogenic activities (DeMartino et al., 2017; Shen et al., 2016). In addition to being harmful to terrestrial and aquatic life, this compound is known to be harmful to humans (Bartholomaeus and Haritos, 2005; Guo et al., 2016; Prudence et al., 2022). For example, as indicated by Shen et al. (2016), the permissible limit for CS_2_ release has decreased from 10 ppm to 1 ppm in the US and Canada. Tailings dams, where gangue and flotation reagent residues are typically discharged, are usually situated in remote areas, with minimal risk of public exposure, either via ambient air or contamination of potable water (NOHSC, 2000). In Europe, several mining sites are situated relatively close to urbanized areas, including near river tributaries to the Meuse and Rhine Rivers (EEA, 2023). Furthermore, it is not clear how much is being released to nearby environment, nor their downstream presence (Yuan et al., 2023).

## Outlook

Xanthates play an essential role in the sectors of the mining and mineral processing industry, metal removal and recovery from wastewater, agriculture, metal protection, rubber vulcanization, pharmaceutical industry, and medicine. Despite the current regulatory developments, scientific literature on xanthates and derivates is scarce. Furthermore, there is a large knowledge gap about xanthates and its wider impacts in European water bodies due to upstream mining activities (Fischer, 2023). Considering the ongoing efforts of electrification and transitioning to sustainable energy systems in response to climate change and economic impetus, metal extraction is not expected to dwindle (Jasansky et al., 2023). For example, phasing-out of lignite is still proving hard to achieve (Radtke and David, 2024). Furthermore, a recent wide European call has been made to scale up domestic mining, processing, and recycling capacity for crucial raw materials (antwerp-declaration.eu). These developments warrant critical need to monitor and manage any by-effects on health and the environment. Xanthates degradation, under different mechanisms and factors, results in the generation of various toxic substances, mainly carbon disulfide (CS_2_), carbonyl sulfide (COS), hydrogen sulfide (H_2_S), and hydrogen peroxide (H_2_O_2_) which are likely to magnify their adversity to humans, animals, soil and aquatic living system. Most (non-)selective analytical methods have been developed in the 1990s with many limitations remaining, thus warranting the advancement of accurate (in)direct xanthate quantification methods (Suvela, 2023). It must be noted that in addition to xanthates, other mining (flotation) chemicals of potential future interest (e.g., ethoxylated tall oil ester of maleic acid, dithiophosphates, thionocarbamates, polyacrylamides, ether diamine, phosphoric acid esters) may also be used depending on the upcoming needs of the mining sector (Fischer, 2023). Based on physicochemical properties and current evidence, xanthates may potentially classify as persistent, mobile and toxic. We call for further investigations to be pursued on the emission permits, use, occurrence, and potential impact of xanthates use by mining activities near transboundary waters on the quality of drinking water sources. In fact, the outcomes and relevance of these investigations could have far more reach than only locally and regionally, considering that half of the world's mining areas are undocumented (Maus and Werner, 2024).

## Conclusions

• Xanthates are versatile compounds with various industrial applications; however, lack of monitoring data raises concern over their occurrence in the environment.
• The current absence of xanthate data in the monitoring databases hampers comprehensive risk assessments, necessitating immediate attention through water sampling and chemical analysis to fill this data gap.
• Recent adoption to include xanthates on the EU watchlist for compounds in surface water will underscore the environmental significance of xanthates, reflecting growing awareness of their potential toxicity and environmental impact, particularly in areas affected by mining activities.
• Calls for exploratory studies on xanthate impact on water quality in Europe signal a need for localized investigation with potential global implications, considering the lack of documentation in many mining and paper industrial areas worldwide.

## CRediT authorship contribution statement

**Daniel J. Duarte:** Writing – review & editing, Writing – original draft, Project administration, Investigation, Conceptualization. **Renske P.J. Hoondert:** Writing – review & editing, Investigation, Conceptualization. **Elvio D. Amato:** Writing – review & editing, Conceptualization. **Milou M.L. Dingemans:** Writing – review & editing, Conceptualization. **Stefan A.E. Kools:** Writing – review & editing.

## Declaration of competing interest

The authors declare that they have no known competing financial interests or personal relationships that could have appeared to influence the work reported in this paper.

## Data Availability

No data was used for the research described in the article
